# Does dipyridamole-induced ischaemia affect NT-proBNP secretion?

**Published:** 2007

**Authors:** Jacques De Greef, Radha Govender, William Vermaak, Nalini Perumal, Mboyo-Di-Tamba Vangu, Elena Libhaber

**Affiliations:** Department of Chemical Pathology, University of Pretoria, Pretoria; Department of Chemical Pathology, University of Pretoria, Pretoria; Department of Chemical Pathology, University of Pretoria, Pretoria; Department of Nuclear Medicine, Johannesburg and Chris Hani−Baragwanath Hospitals, University of the Witwatersrand, Johannesburg; Department of Nuclear Medicine, Johannesburg and Chris Hani−Baragwanath Hospitals, University of the Witwatersrand, Johannesburg; Chris Hani−Baragwanath Hospital, University of the Witwatersrand, Johannesburg

## Abstract

**Background:**

B-type natriuretic peptide (BNP) and the inactive amino-terminal pro-B-type natriuretic peptide (NT-proBNP) have a prognostic value in heart failure and in myocardial infarction. There has been some evidence that BNP and NT-proBNP can be used in the diagnosis of myocardial ischaemia by improving the sensitivity of exercise-stress testing.

**Objective:**

To observe the relationship between dipyridamole-induced ischaemia and the secretion of NT-proBNP. Methods: A total of 52 consecutive patients, referred for dipyridamole stress ^99m^Tc-sestamibi single-photon emission computed tomography (SPECT) myocardial perfusion imaging (MPI) to diagnose reversible ischaemia, were enrolled. NT-proBNP was determined at rest and one hour after infusion of dipyridamole.

**Results:**

Of the 52 patients, 25 had normal scans, 12 had scans with fixed defects (previous myocardial infarction with no inducible ischaemia) and 15 had reversible perfusion defects (inducible ischaemia). There was no correlation between ischaemia and resting NT-proBNP, post-stress NT-proBNP and the change in NT-proBNP. There was a correlation between ejection fraction, ventricular volumes and resting NT-proBNP.

**Conclusions:**

NT-proBNP does not add to the diagnosis of myocardial ischaemia in dipyridamole stress MPI.

## Summary

The cardiac natriuretic peptides (CNP) have been shown to be of value in the diagnosis, prognosis, treatment and monitoring of heart failure.^1^-^4^ There have been studies showing an increase in BNP and NT-proBNP due to exercise in non-ischaemic5 and ischaemic individuals.^6^-^12^ This increase was greater in patients with myocardial ischaemia. The use of BNP has even been proposed to improve the sensitivity of exercise-stress testing.^9^ In a selected population, NT-proBNP had a sensitivity of 90% in predicting myocardial ischaemia; this was considerably higher than that of the stress ECG (37%). In a further study where the subjects were not selected, these observations were confirmed.^11^ However, the contribution of BNP during exercise in the diagnosis of myocardial ischaemia was less impressive. Not only was there an increase in BNP in these patients due to stress, but there was also a higher baseline value in the ischaemic compared to the non-ischaemic group.

Myocardial ischaemia can be induced using physical or pharmacological stress. Dipyridamole is often used in the setting of MPI. Dipyridamole has been shown to induce perfusion and regional wall motion abnormalities. The mechanism of this regional redistribution of blood flow is probably multifactorial. A proposed mechanism is due to steal, whereby the normal vessels vasodilate, with resultant enhanced blood flow, leaving relatively reduced pressure and flow across areas of haemodynamically important coronary artery stenoses, inducing ischaemia and regional wall motion abnormalities. Another proposed mechanism is that due to the afterload reduction of dipyridamole, there is a degree of hypotension and reflex tachycardia and this causes or contributes to the myocardial ischaemia.^13^

Dipyridamole-induced myocardial ischaemia has also been shown to increase atrial natriuretic peptide (ANP),^14^ however the exact role of the natriuretic peptides in the diagnosis and management of myocardial ischaemia still needs to be elucidated.

There are many terms referring to the natriuretic peptides. Pro-BNP (108 AA) is cleaved by a serine protease corin into an inactive protein pro-BNP 1-76 (this is also referred to as NT-proBNP and is the analyte measured by the pro-BNP assay from Roche), and into BNP 32, the active hormone, also referred to as BNP. BNP and NT-proBNP can reliably be measured *in vitro*.^15^-^17^ BNP has a half-life of 19 minutes, whereas NT-proBNP has a half-life of 90 minutes. NT-proBNP has been shown to be as effective in diagnosing left ventricular (LV) dysfunction as BNP,^18^ and the longer half-life makes it easier to use in the clinical setting.

The aim of this study was to observe whether dipyridamole-induced myocardial ischaemia caused a predictable change in NT-proBNP values, and whether these changes could be used to diagnose myocardial ischaemia.

## Material and methods

Fifty-two consecutive patients referred for dipyridamole stress SPECT MPI at the Nuclear Medicine Department of the Johannesburg Hospital to exclude reversible ischaemia, were enrolled into the study. The study had institutional ethics approval, and informed consent was obtained from the subjects prior to enrolment. The study period was between January and October 2006. The median age was 63 years (40−83); the male-to-female ratio was 26:26.

The exclusion criteria were: asthma or severe chronic obstructive pulmonary disease, beta-blocker use, the intake of caffeine in the 24 hours preceding the test, and refusal of consent. There were no adverse reactions to dipyridamole during the time of the study.

## SPECT MPI protocol

A two-day ^99m^Tc-methoxyisobutyl isonitrile (Sestamibi) SPECT MPI protocol was used. On the first day the stress test was performed, with the rest study being done on day two. Dipyridamole and ^99m^Tc sestamibi (740 MBq) were given according to protocol. The patient was allowed to rest and images were acquired between 30 and 60 minutes after the injection of the radiotracer. Imaging was performed using a double-head rotating field-of-view gamma camera (Infinia Hawkeye, GE Medical Systems) equipped with low-energy, high-resolution, parallel-hole collimators. SPECT images were acquired on a 64 × 64 matrix using the step-and-shoot mode with a total of 180-degree angular range and 20 seconds per image.

## MPI assessment

Myocardial ischaemia was assessed visually and by means of semi-quantitative scoring of the extent and severity of perfusion abnormality − summed difference score (SDS). This was calculated by subtracting the summed resting score (SRS) from the summed stress score (SSS), using the xeleris multisync work station for processing data, which was fitted with the QGS/QPS and uses a 20-segment model to quantify myocardial perfusion on an arbitrary scale. The sensitivity of the SPECT MPI technique in diagnosing ischaemic heart disease is 91% and the specificity is 81%,^19^ compared to exercise stress ECG with a sensitivity of 37% and a specificity of 83%.^9^ Three trained nuclear physicians performed the MPI assessments; they were blinded to the NT-proBNP results.

An SDS of more than four indicates the presence of myocardial ischaemia. This can be graded: an SDS of 4–8 is mild, 9–12 is moderate and greater than 13 is severe myocardial ischaemia. If there was any doubt about the significance of the myocardial ischaemia, the visual inspection of the MPI study was used to assess the significance of the perfusion defect.

## Blood sampling

The reported timing of blood sampling for NT-proBNP is in subjects undergoing physical stress testing and in some cases where BNP is analysed in conjunction with NT-proBNP. BNP has a half-life of 19 minutes and this forms the rationale for early blood sampling when BNP is the analyte under investigation. NT-proBNP has a half-life of 90 minutes. These differing half-lives have an effect on optimal timing of blood sampling; it cannot be extrapolated that an optimal time for BNP would be optimal for NT-proBNP. A further factor to consider is that even though dipyridamole has a maximal cardiac effect after five minutes of infusion, this effect persists for 10 to 30 minutes after infusion.^20^ For this reason, blood sampling was performed at various times to obtain the optimal timing for NT-proBNP sampling.

**Fig. 1.**
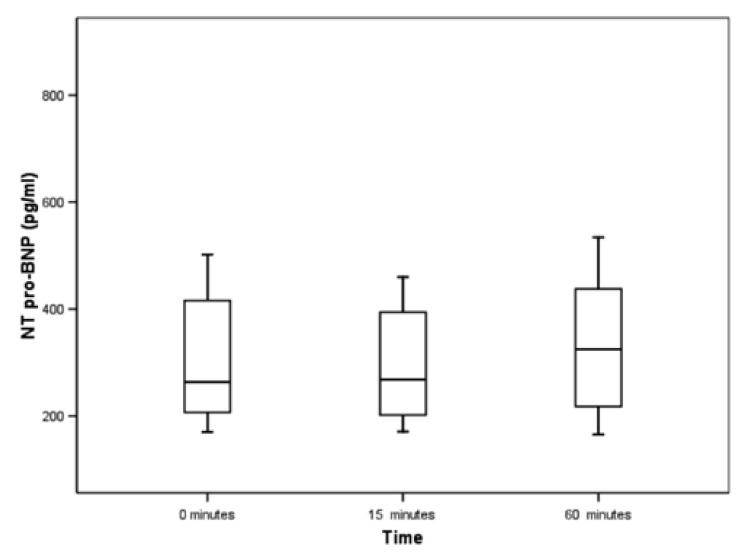
Timing of NT-proBNP.

Blood samples were obtained at rest, 15, 30 and 60 minutes after the infusion of dipyridamole in 15 subjects. The maximal change was observed at 60 minutes. The experiment was repeated on 10 more subjects to confirm this observation, only sampling at 15 and 60 minutes (Fig. 1). This was the rationale for a resting and a one-hour blood sample.

A resting blood sample was obtained before the dipyridamole infusion and the post-stress blood sample was obtained one hour after the infusion in all subjects. The sample was allowed to clot and then centrifuged at 3 000 rpm for 10 minutes. The serum was stored at –70°C and analysed in batches.

## NT-proBNP assay

Roche Diagnostics SA supplied the assay, which is an electrochemiluminesence immunoassay that uses a sandwich technique. The analytical range is 5−35 000 pg/ml; the reference cut-off value based on the manufacturer’s data is 125 pg/ml. Patients with values below this exclude cardiac dysfunction with a high level of certainty, levels above 125 pg/ml may indicate cardiac dysfunction and this is associated with an increased risk of cardiac complications (myocardial infarction, heart failure, death). This value is not corrected for age or gender (women tend to have higher values than men and values increase with age). The analytical coefficient of variation for the assay was 1.8−2.7%.

## Statistical analysis

This was done using SAS Software V9.1 (SAS Institute Inc, Cary, NC, USA). Spearman correlation coefficient was used to calculate correlations, and the Kruskal−Wallis test was used to calculate differences in NT-proBNP between the groups.

## Results

Of the group of 52 patients, 25 had a normal scan (no perfusion defects), 12 had a fixed perfusion defect (a previous infarction, but no reversible ischaemia) and 15 had a reversible perfusion defect (inducible ischaemia). Of the 15 patients with reversible defects, five had fixed defects as well (Table 1).

**Table 1:** Study Group Characteristics

	*Normal*	*Fixed defect*	*Reversible defect*
Number of subjects: total	25	12	15
Hypertension	5	4	9
Known IHD	5	5	4
Chest pain	7	7	5
Diabetes mellitus	3	1	3
Pre-operative	9	3	2
Median NT-proBNP (pg/ml) (range)	236 (51−8840)	386 (28−16 334)	330 (6−4151)
Median EF (%) (range)	58 (18−78)	37.5 (8−64)	58.5 (18−82)

IHD: ischaemic heart disease; EF: ejection fraction; NT-proBNP: N-terminal pro-B-type natriuretic peptide.

**Fig. 2.**
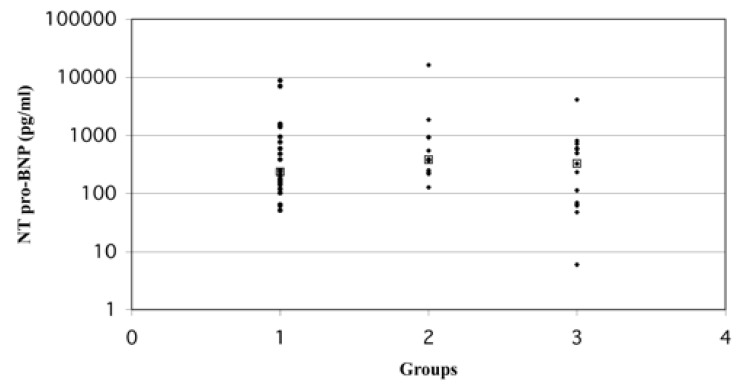
The patient groups found in the study population. 1 = normal (no perfusion defects) (*n* = 25) median = 236 pg/ml; 2 = fixed perfusion defect (no inducible ischaemia) (*n* = 12) median 5 386 pg/ml; 3 = reversible perfusion defect (inducible ischaemia) (*n* = 15) median = 330 pg/ml.

The median resting NT-proBNP of all the subjects was 349 pg/ml, with a range of 6−16 334 pg/ml. The median resting NTproBNP in patients with normal scans was 236 pg/ml, for those with fixed defects, 386 pg/ml, and for patients with reversible defects, 330 pg/ml. The highest median resting NT-proBNP result was in the subjects with a fixed perfusion defect, followed by the subjects with reversible ischaemia, and the lowest was seen in the subjects with normal MPI scans. There was a large overlap of values in the different subject groups; these differences are not statistically significant (Fig. 2).

**Fig. 3.**
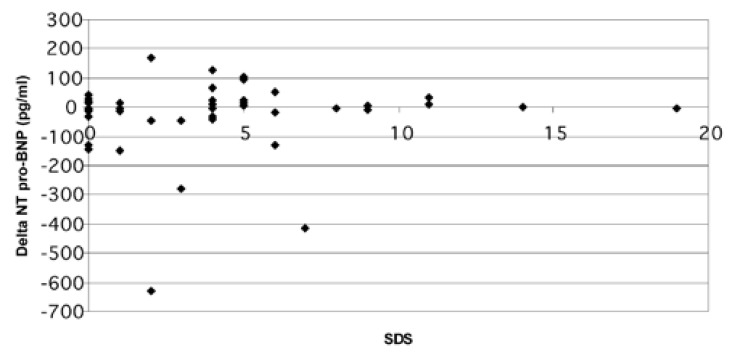
The relationship between the degree of ischaemia (SDS) and delta NT-proBNP (post-stress NT-proBNP – resting NT-proBNP). SDS: summed difference score.

**Fig. 4.**
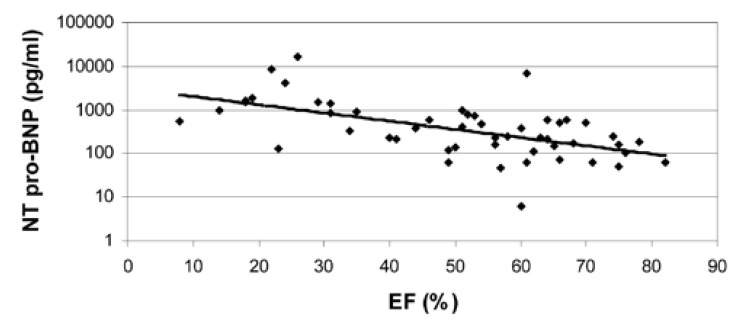
The relationship between NT-proBNP and ejection fraction (EF) (%).

**Fig. 5.**
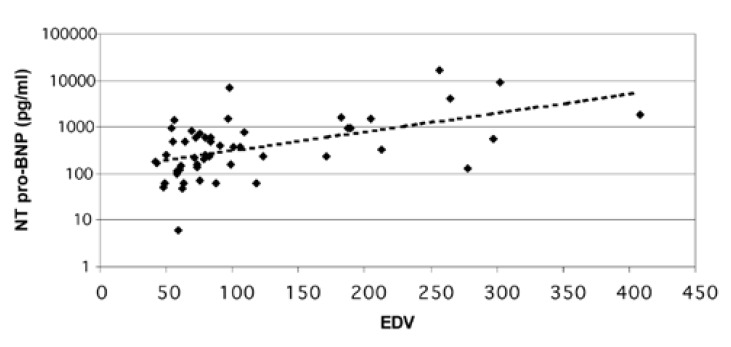
The relationship between NT-proBNP and end-diastolic volume (EDV).

There was no correlation between the ischaemic index (SDS) and resting, post-stress and delta NT-proBNP values (Fig. 3). There was a strong correlation between NT-proBNP and resting LV ejection fraction (*r* = 20.58, *p* < 0.0001), LV end-systolic volume (*r* = 0.58, *p* < 0.0001) and LV end-diastolic volume (*r* = 0.54, *p* = 0.0002) (Figs 4, 5).

## Discussion

This study used a sample population that was made up of the typical patient referred for dipyridamole stress SPECT MPI to assess the presence of reversible myocardial ischaemia. No selection was made on the basis of ventricular function.

The subjects in this study population probably had a degree of LV dysfunction, as demonstrated by the median resting NT-proBNP of 349 pg/ml (N cutoff 5 125 pg/ml). The correlation seen between NT-proBNP and LV ejection fraction, LV enddiastolic and LV end-systolic volumes was a reflection of the relationship of NT-proBNP with ventricular function.

In the exercise studies, where NT-proBNP increased significantly in individuals with myocardial ischaemia, these individuals were selected and had normal ejection fractions.^9^ However, when an unselected population was used, the contribution that BNP made to the diagnosis of myocardial ischaemia in exercisestress testing was statistically significant but weak.^11^ In some studies the change in BNP was not diagnostic of myocardial ischaemia, but the baseline BNP was diagnostic/predictive of myocardial ischaemia.^8^,^11^,^21^ This suggests that ventricular function may play a role in the observation that exercise-induced myocardial ischaemia causes an increase in cardiac natriuretic peptide (CNP) secretion. It also suggests that the relationship between exercise-induced myocardial ischaemia and CNP secretion is not fully elucidated.

The mechanism of inducing myocardial ischaemia may also be important. The effect that physical exercise has on CNP has been studied. The effect of pharmacological stress testing has not been investigated to the same extent. There has been one study demonstrating an increase in atrial natriuretic peptide with dipyridamole-induced myocardial ischaemia.^14^ In dobutamine-induced myocardial ischaemia there was no correlation between myocardial ischaemia, as diagnosed with echocardiography as wall motion abnormalities, and the secretion of BNP. There was, however, a correlation between resting BNP and myocardial ischaemia.^22^-^24^

There is a debate as to which marker to use, BNP or NT-proBNP. These markers are secreted in equimolar amounts. The metabolism of NT-proBNP is not fully understood and renal failure is speculated to influence the values. In studies on the use of these markers there is little to choose between them and either can be used.^18^ Whichever marker is offered by the local laboratory would be acceptable. The only issue is that of the differing half-lives, as this will influence the timing of blood sampling in dynamic studies.

## Conclusions

In this study, using SPECT MPI with a sensitivity of 91% to diagnose myocardial ischaemia, there was no correlation between myocardial ischaemia and NT-proBNP – resting values, post-stress values or the change in values. The small number of ischaemic subjects may have limited this study and a larger study may discover statistically significant findings. The question still remains whether NT-proBNP can be used to diagnose myocardial ischaemia in the individual patient presenting with suspected myocardial ischaemia.

The median NT-proBNP values were higher in the subjects with fixed defects and inducible defects compared to the subjects with normal perfusion scans. However, there was enormous overlap between the values, making the resting NT-proBNP value useless to predict ischaemia. Apart from the prognostic value of NT-proBNP, it has no value in the diagnosis of myocardial ischaemia in the typical patient referred for dipyridamole stress SPECT MPI.
